# An updated scoping review of migrant health research in Ireland

**DOI:** 10.1186/s12889-024-18920-0

**Published:** 2024-05-28

**Authors:** Anne Cronin, Ailish Hannigan, Nuha Ibrahim, Yuki Seidler, Blessing Olamide Owoeye, Wigdan Gasmalla, Tonya Moyles, Anne MacFarlane

**Affiliations:** 1 School of Medicine, University of Limerick, Plassey Park Road, Castletroy, Co., Limerick, V94T9PX Ireland; 2 University of Vienna, Vienna, Austria; 3 Cairde, Dublin 1, Ireland

**Keywords:** Migrant, Refugee, Health, Scoping review, Ireland, World health organisation

## Abstract

**Background:**

One in five people living in Ireland is a migrant. Understanding the distinctive health needs of this diverse population is essential to provide evidence-based, culturally sensitive primary care services. The aim of this review is to systematically examine changes in migrant health research in Ireland and to inform research, policy and practice in the field.

**Methods:**

To update a 2017 scoping review of migrant health research in Ireland, we used Arksey and O’Malley’s framework, updates by Colquhoun and Peters and the PRISMA-ScR from the Joanna Briggs Institute to search 10 databases covering May 2017 - March 2023. Findings were analysed using the World Health Organisation Strategy and Action Plan for Refugee and Migrant Health 2016–2023, which identifies 9 priority strategic areas (SA). Findings were compared with the 2017 review.

**Results:**

62 papers were identified. There has been an increase in studies over time from an average of five per year in the previous review to an average of 10 per year in this review. There is growing interest in research about *SA1: Collaborative action on migrant health issues* and *SA2: Advocacy for the right to health of refugees and migrants* – evidenced by an increase of 13% in this review. Similarly to 2017, the majority of papers align with three of the nine WHO Strategic Areas; *SA3: Addressing the social determinants of health* (24%), *SA4: Achieving public health preparedness* (29%) and *SA5: Strengthening health systems* (26%). The volume of research on *SA6: Communicable diseases* (11%) and *SA7: Noncommunicable diseases* (19%) remains stable however research on *SA8: Health screening and assessment* (5%) and *SA9: Improving health information and communication* (2%) remains low.

**Conclusions:**

The increase in the volume of research on migrant health in Ireland is notable. The analysis over time illuminates changes in the focus of research studies. Gaps in research about screening, assessment and health information warrant particular attention. It is also necessary to continue paying attention to areas of recent growth and stagnation for a balanced and comprehensive evidence base. Mobilising resources to continue this increase is needed for evidence-based policy and practice.

**Supplementary Information:**

The online version contains supplementary material available at 10.1186/s12889-024-18920-0.

## Background / Introduction

Globally, one in every eight people or 13% of our global population is either a migrant or has been forcibly displaced by conflict, persecution, climate crisis, poverty, or the lack of security and opportunity [1, 2].

There is no universally accepted definition of migrant. However, a broad and widely used definition from the International Organisation for Migration is that migrant is;

*‘An umbrella term, not defined under international law, reflecting the common lay understanding of a person who moves away from his or her place of usual residence, whether within a country or across an international border, temporarily or permanently, and for a variety of reasons* [3].

This broad definition also includes refugee as;

*‘A person who, owing to a well-founded fear of persecution for reasons of race, religion, nationality, membership of a particular social group or political opinion, is outside the country of his nationality and is unable or, owing to such fear, is unwilling to avail himself of the protection of that country* [3].

This review focuses specifically on international migrants i.e., people who live in the Republic of Ireland (hereinafter referred to as “Ireland”) as a refugee or migrant, having moved to Ireland from another country or from the country in which they were born. We use the terms refugee and migrant in line with the WHO Strategy and Action Plan (WHO SaAP) for Refugee and Migrant Health in the WHO European region 2016–2022 [4] which is used to inform our analysis (described in more detail later).

The World Health Organisation (WHO) Global Research Agenda on Health, Migration and Displacement identifies priority areas for action and emphasises the importance of setting out country-specific priorities and documenting country-level actions, which is essential to promote progress through sharing and learning [5]. Therefore, it is important to know what evidence is available on the health of migrants in different country settings. There are examples of evidence reviews in several countries, including the United Kingdom, Norway, Canada, the European Union (EU) and Ireland, which is the focus of this paper [6–9].

According to the most recent Census of Population of Ireland in 2022, 20% of the resident population was born in another country. This represents over a million people, an increase of 207,031 from 2016. The biggest increases were in the number of people born in India, Brazil and Romania. In the twelve months to April 2023, over 80,000 non-Irish citizens moved to Ireland, a 16-year high [10].

Factors contributing to higher migration rates include employment opportunities in Ireland due to strong economic performance [11]. The latest Eurostat data indicates that Irish people are two-times better off than the average EU citizen based on purchasing power parities and Gross Domestic Product (GDP) per capita [12]. Opportunities to study, family reunification programmes and Ireland’s reputation as one of the richest countries in the EU also contribute.

Separately from the census figures, approximately 100,000 Ukrainian refugees were receiving temporary protection from the Irish government as Beneficiaries of Temporary Protection (BoTP) and 26,092 people from other countries were receiving International Protection (IP), at the end of 2023. These figures exclude approximately 4,500 refugees coming from countries such as Syria and Afghanistan receiving protection through the Irish Refugee Protection Programme [13].

Ireland has produced two intercultural health strategies to optimise refugees and migrants’ access to healthcare and their health outcomes; the National Intercultural Health Strategy (NIHS) 2007–2012 [14] and the Second NIHS 2018–2023 [15]. Since 2014, the NIHS is aligned with the Public Sector Equality and Human Rights Duty, which places a legal requirement on all public bodies to comply with Sect. 42 of the Irish Human Rights and Equality Act 2014 [16].

## Migration and health in Ireland

Unlike many other EU countries, primary care in Ireland is not universal and incurs a cost. This can make healthcare unaffordable and consequently inaccessible [17]. New entrants to Ireland are covered by public health services (some of which are free) when they have been living in the country for one year or intend to live in the country for one year. This is called being ‘ordinarily resident’. For those seeking refugee status, the means of accessing and receiving healthcare can vary considerably depending on the level of state protection provided which influences whether or not they are considered ordinarily resident. For example, in Ireland people seeking international protection can apply for refugee status, subsidiary protection or temporary protection. The affordability of healthcare is shaped by these categorisations: each category has different implications for eligibility to a social support and, therefore, access to free general practice care or not [18, 19]. For example, persons seeking asylum / international protection receive €38.80 per week for adults and €29.80 per week for children (if housed in state provided accommodation). They also receive a medical card which entitles them to free GP access However, individuals granted refugee status, subsidiary protection, or leave to remain have access to social welfare on par with Irish citizens. This involves a bureaucratic process that is far more complex than in other States [20].

Whilst eligibility criteria may present difficulties for some, impediments to healthcare access arising from communication barriers introduce separate challenges. The 2022 census data highlights that 751,507 people spoke a language other than English or Irish at home, representing an increase of 23% from 2016; 11% of whom indicated that they did not speak English well and 2% did not speak it at all. Accordingly, people with limited English-language proficiency accounted for 1.9% (*n* = 97,695) of the overall Irish population [10]. Ireland has low availability of trained interpreters [21] and this compromises communication in healthcare consultations, thus undermining a vital component of accessible healthcare [22, 23].

## Refugee and migrant health research in Ireland

**Fig. 1:**
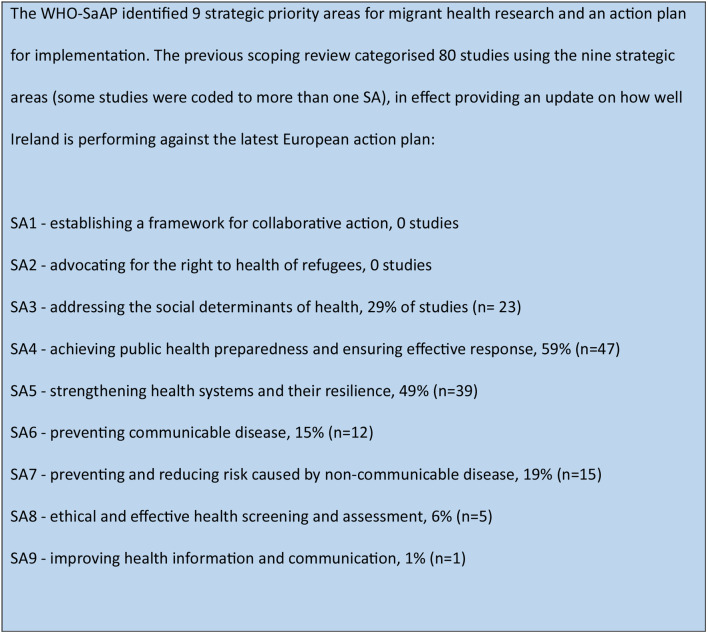
Results from migrant health research in the republic of Ireland: a Scoping review [26]

There have been three evidence syntheses of refugee and migrant health research in Ireland. Two were in the early 2000s [24, 25]. The third and most recent information mapped the scientific literature on migrant health in Ireland, through research studies published between 2001 and 2017 [26]. The findings of the 2017 review were analysed using theWHO-SaAP for Refugee and Migrant Health in the WHO European region 2016–2022 [4]. As presented in Fig. 1, the 2017 review found that while strategic areas (SA) related to the social determinants of health (SA 3), public health preparedness (SA 4) and strengthening health systems (SA 5) have been well-researched, there were gaps in research about collaborative action (SA 1), advocacy and human rights (SA 2), screening (SA 8), and health information systems (SA 9), with a recommendation for more inter-disciplinary projects. The authors found that almost one in five studies did not have a primary focus on migrant health but contributed information on the topic through an analysis of data by, for example; country of birth, nationality or ethnicity.

Our review is an update of Villaroel et al., covering the period May 2017 to March 2023.

The rationale for updating the previous scoping review is four-fold. First, there have been significant changes in migration patterns in the EU and Ireland over the past six years (2017–2023). Second the pace of publication in the area of migrant health has increased significantly in an international context, and the authors wanted to investigate whether the pace had similarly increased in an Irish context. Thirdly, the National Intercultural Health Strategy 2019–2023 [15] has come to an end and Irish policy makers need the most up-to-date evidence to guide new strategic developments. Finally, the WHO-SaAP 2016–2022 [4] has also ended and it is therefore timely to conduct an updated review of the literature for this period to analyse the congruence between Irish evidence and the WHO strategic areas over time. The current Action Plan for Refugee and Migrant Health in the WHO European Region 2023–2030 was adopted in October 2023 [27].

Thus, the aim of this scoping review is to update the work completed in 2017 by incorporating the most recent contributions to migrant health research in Ireland conducted between 2017 and 2023.

## Methodology

**Fig. 2:**
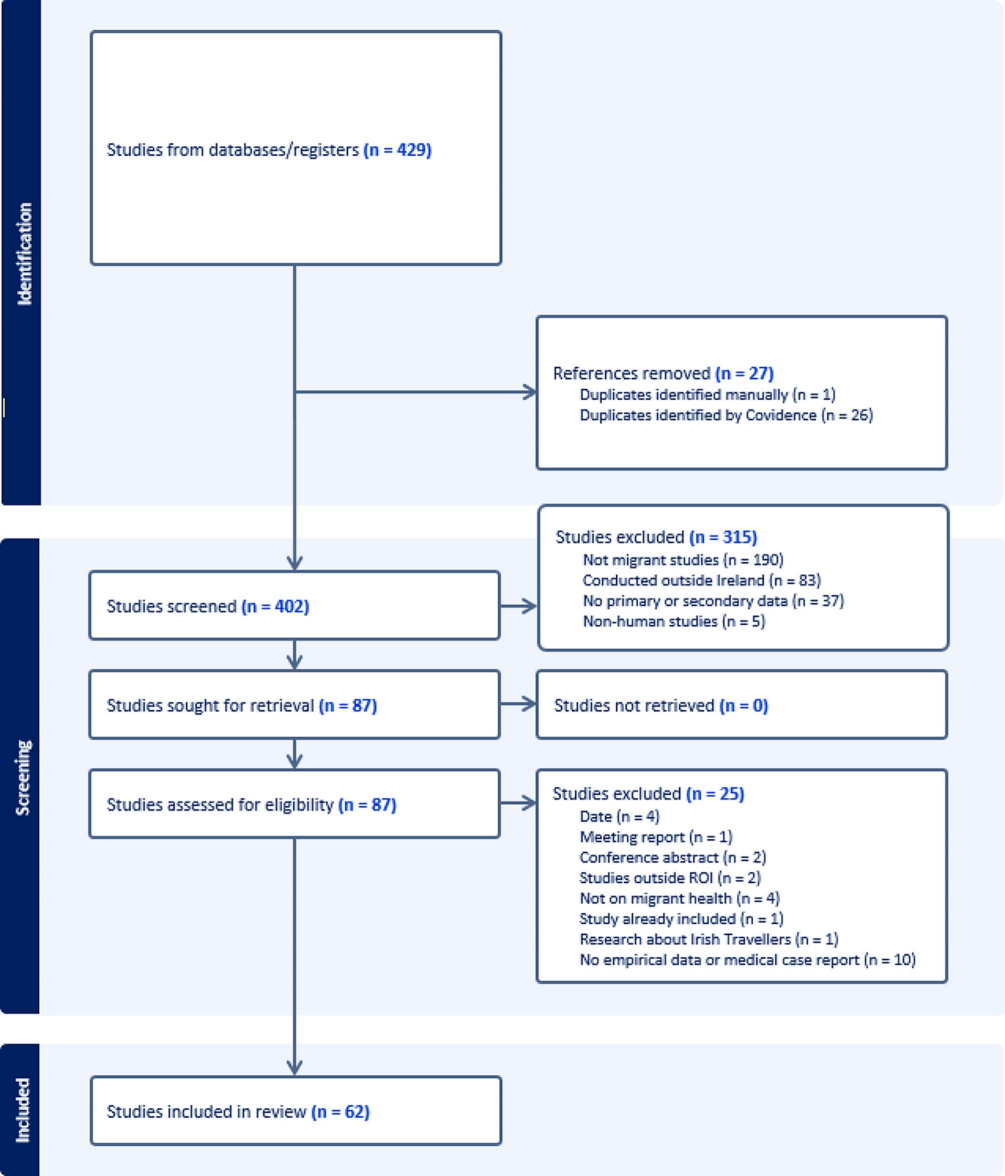
PRISMA Flow Chart

The current scoping review’s methodological approach was guided by Peters [28] and Levac and Colquhoun [29] building on Arksey and O’Malley’s 6 -stage framework [30]. The work was conducted using the Joanna Briggs Institute (JBI) Manual for Evidence Synthesis [31] and is reported according to the Preferred Reporting Items for Systematic Reviews and Meta-Analyses extension for Scoping Reviews (PRISMA-ScR) recommendations (Fig. 2). The protocol is registered with Open Science Framework (OSF) Registries (Cronin, A., Ibrahim, N., MacFarlane, A., Hannigan, A., & Seidler, Y. (2023, May 2). Updated Scoping Review of Migrant Health Research in the Republic of Ireland. 10.17605/OSF.IO/2KGMH).

### Stage 1 identifying the research question

The research question remained consistent with the research question developed in 2017; ‘What is the scope, main topics and gaps in evidence in the existing literature on health of migrants residing in the Republic of Ireland?’ The population is refugees and migrants, the context is Ireland, and the concept is research on migrants.

### Stage 2 identifying relevant studies

Congruent with the original scoping review, we conducted systematic searches of 10 electronic databases; PsycINFO, Psych Articles, CINAHL, Medline, Academic Search Complete, Cochrane Library, Embase, Web of Science, Econlit and Lenus. Social Sciences Full Text (H.W. Wilson) was no longer available. The research team actively collaborated with the University of Limerick librarian to formulate a search strategy and pilot it across all databases to optimise the search process and to ensure all relevant studies were included. The final search terms are consistent with the previous scoping review and set out in Table 1.

**Table 1:** Search terms

Population	asylum* OR refugee* OR migrant* OR migrat* OR emigrant* OR emigrat* OR immigrant* OR nomad* OR foreign* OR ethnic* OR displaced OR stateless OR state-less OR noncitizen* OR non-citizen* OR outsider* OR newcomer* OR “newly arrived” OR “new arrival*” OR “recent entrant*” OR “non national” OR non-national
Context	Republic of Ireland, Ireland, Irish
Concept	Health

Given the volume of peer-reviewed literature available, the original scoping review did not include grey literature. For comparability of methods, we also excluded grey literature in this updated review.

The final and complete search was conducted on the 10th of April 2023.

### Stage 3 study selection

Articles were included if the empirical research was based on primary or secondary data on the health of migrants in the Republic of Ireland; peer-reviewed publications; and articles in the English language. Since our aim was to map new knowledge against the knowledge synthesised from the first review, the timeframe was limited to articles published from May 2017 to March 2023 only.

Similar to the original scoping review, we also included studies that were not primarily focused on migrant health but collected data on, for example, country of birth, ethnicity, nationality, or citizenship and reported on these subgroups in their analysis of the data. We also included multi-country studies where Irish data was identifiable.

### Stage 4 data charting

We used EndNote to manage retrieved articles from the ten databases and exported the articles to the screening tool Covidence. There were two pairs of reviewers, (AC and WG / YS and BO). Each title and abstract was independently screened by a pair of reviewers who then compared their decisions. Any disagreement was resolved by the larger group (AC, YS, WG, BO) and recorded reasons for exclusion. Once title and abstract screening was complete, the research team met bi-weekly to conduct a full text screening of the remaining articles (AC, YS, WG, BO).

Data were extracted from the studies (WG, BO) and the process reviewed, discrepancies and conflicts examined and final consensus agreed by AC and YS, who ensured the completeness and accuracy of the data extraction process. The data extraction sheet was designed in an excel format applying the same headings used in the original review; authors, publication year, title of the study, geographic location of the study, data collection period, study design, target population, target migrant group, definition of migrant group, participant group, study objective(s), data collection methods, and main study findings. However, we also included additional headings to identify whether studies with a secondary focus on migrant health analysed the demographic data on ethnicity / country of origin that they collected.

As with the original review, we carried out a quality assessment of the studies to ensure consistency and to add an extra layer of academic rigour (see supplementary file). The critical appraisal tools used include the updated Critical Appraisal Skills Programme (CASP) for qualitative studies [32]; Guidance for Reporting Involvement of Patients and the Public (GRIPP 2) for studies that reported on the involvement of migrants in their research process [33]; the Mixed Methods Appraisal Tool (MMAT) for mixed-methods studies [34]; the AXIS tool for cross-sectional studies [35]; the Newcastle Ottawa Scale for cohort studies [36]; the JBI checklist for diagnostic test accuracy studies [37] and the JBI critical appraisal tool for case series [31].

### Stage 5 collating, summarising and reporting results

The team met to reach consensus on the coding process and met again once half of the papers were coded to test consistency and discuss any modifications (the coding rules are included in supplementary files). Studies were coded under the nine WHO strategic areas (see Fig. 1). Each study was matched with the corresponding strategic area based on its primary aim. In cases where a study had a dual focus, it was mapped to two relevant strategic areas. We documented health topic separately e.g. sexual and reproductive health, mental health, antenatal care.

### Stage 6 consultation with stakeholders

In line with updated guidance on consultation with stakeholders as a required stage in scoping reviews [38], we consulted with a community partner for two purposes: first, to review and interpret findings in the context of their experience and second, to support knowledge transfer to the NGOs and migrants living in Ireland as well as policy makers that they meet through their advocacy work.

The community stakeholder (TM) is the general manager of Cairde, a community health development organisation in Dublin, with over 20 years’ experience of working with refugees and migrants. TM is a co-researcher on migrant health projects conducted with some members of the research team. TM was invited to participate and contribute to the interpretation of findings in a one-to-one meeting with AC. TM’s contribution was integrated into the write up of the results and discussion sections of this paper to present our combined interpretation and synthesis of the scoping review findings. In addition, some specific insights or considerations from the perspective of someone working in an NGO focused on refugee and migrant health are made explicit in the Discussion.

As a co- author, TM has contributed to drafting and approval of the final manuscript.

## Results

A Preferred Reporting Items for Systematic reviews and Meta-Analyses extension for Scoping Reviews (PRISMA-ScR) flow diagram illustrating the process is outlined in Fig. 2. The completed PRISMA-ScR checklist is included in supplementary files.

### Overall findings

429 articles were identified from the 10 databases and after removing 27 of them for duplication, 402 remained for title and abstract screening. We conducted title and abstract screening and recorded reasons for exclusion; 60% were not migrant studies (*n* = 190), 26% were conducted outside Ireland (*n* = 83) and 12% didn’t collect any primary or secondary data (*n* = 37). Less than 1% (*n* = 5) were excluded due to date of publication or non-human studies. We conducted full text screening on the remaining 87 articles and excluded a further 25 articles for reasons including; no empirical data on health of migrants (40%, *n* = 10); not a migrant study (16%, *n* = 4) or the date was outside the inclusion criteria (16%, *n* = 4). The final number of included studies is 62, the characteristics of which are listed in Table 2.

**Table 2:** Summary of included articles

Items ID	Authors	Year	Study objectives	Primary Focus Y/*N*	How being a migrant was defined	National /International	Study design	Health topic	WHO SaAP Strategic Area
1	Azvee Z, et al.	2021	Migratory trend of mental health professionals in Ireland	N	Citizenship	Ireland	Quantitative	Mental health	3
2	Barlow P, et al.	2022	Healthcare utilisation of immigrants	Y	Nationality	Ireland	Quantitative	Healthcare utilisation	2
3	Barrett P, et al.	2018	Measles outbreak in Ireland	Y	Nationality / Ethnicity	Ireland	Mixed methods	Communicable disease	6
4	Bogdanet D, et al. (a)	2022	Predicting gestational diabetes mellitus (GDM) at 24 to 28 weeks of gestation.	N	Race	Ireland	Quantitative	Sexual and reproductive health	7
5	Bogdanet D, et al. (b)	2022	Predicting oral glucose tolerance test at 24–28 weeks of gestation	N	Race	Ireland	Quantitative	Sexual and reproductive health	7
6	Brady M, et al.	2020	Voluntary community-based HIV testing	N	Nationality/ Asylum status	Ireland	Quantitative	Screening	6, 8
7	Carroll HK, et al.	2022	Impact of the COVID-19 pandemic on non-EEA doctors working in Ireland	Y	Nationality	Ireland	Quantitative	Communicable disease	5
8	Clarke N, et al.	2017	Predictors of trainee doctor emigration from Ireland.	N	Nationality	Ireland	Mixed methods	Healthcare workers	3
9	Colaceci S, et al.	2023	Italian midwives’ decision to migrate	Y	Nationality	UK, Ireland, Germany, Switzerland, and Spain	Qualitative	Healthcare workers	3
10	Collins C, et al.	2022	Health status of Syrian refugees in Ireland	Y	Nationality	Ireland	Quantitative	Healthcare utilisation	4
11	Cotter S, et al.	2019	Relationship between migration, psychopathology and stressful events in children and adolescents.	Y	Citizenship	Ireland	Quantitative	Mental health	7
12	Cruise SM, et al.	2018	Factors associated with depressive symptoms amongst a sample of mothers of 9-month-old infants in the Republic of Ireland	N	Race	Ireland	Quantitative	Mental health	7
13	Curley A, et al.	2019	Mental capacity of inpatients to make decisions around their care	N	Nationality	Ireland	Quantitative	Mental health	7
14	Duffy RM, et al.	2017	Demographic profile of those that attended Spriasi	Y	Asylum seeker or refugee	Ireland	Quantitative	Mental health	4, 7
15	Finnegan J, et al.	2019	Trends and variances between a racially diverse stroke population	Y	Nationality / Ethnicity	Ireland	Quantitative	Non-communicable disease	7
16	Finnegan R, et al.	2022	TB infection in a Direct Provision centre in Ireland.	Y	Asylum seeker or refugee	Ireland	Quantitative	Communicable disease	3,6
17	Greenwood RM, et al.	2017	Exclusion from ordinary privileges and overt discrimination - experience of visible and non-visible immigrants.	Y	Race	Ireland	Mixed methods	Social determinants of health	2
18	Hannigan A, et al.	2020	National health and social care data collections with information on ethnicity	Y	Ethnicity	Ireland	Quantitative	Health information	9
19	Haran M, et al.	2023	Physical restraints and seclusion in child and adolescent mental health settings	N	Race	Ireland	Quantitative	Mental health	7
20	Harrington S, et al. (a)	2021	Prevalence of congenital CVD in school children and associated socio-demographic factors.	N	Ethnicity	Ireland	Quantitative	Social determinants of health	8
21	Harrington S, et al. (b)	2023	Relationship between time spent on screens and reading/writing with ocular conditions in school children	N	Race / Nationality	Ireland	Quantitative	Social determinants of health	8
22	Harrington SC, et al. (c)	2019	Increasing use of digital media by schoolchildren as a risk factor for ocular diseases.	N	Ethnicity	Ireland	Quantitative	Social determinants of health	3
23	Hoare R.	2020	Football as a lingua franca for unaccompanied minors in Ireland seeking asylum	Y	Asylum seeker	Ireland	Qualitative	Mental health	5
24	Jabakhanji SB, et al.	2018	BMI in young children	N	Nationality / race	Ireland	Quantitative	Child health	4
25	Kelleher D, et al. (a)	2020	Transnationalism influences health preferences and health-related behaviours of Eastern European migrants	Y	Nationality	Ireland	Quantitative	Health utilisation	4
26	Kelleher D, et al. (b)	2022	Polish migrant’s use of GP visits in Ireland and Poland	Y	Nationality	Ireland	Quantitative	Health utilisation	4, 5
27	Kelleher D, et al. (c)	2022	Polish migrants and native Irish differences in health state utility valuations	Y	Nationality	Ireland	Quantitative	Health utilisation	4, 5
28	Kennedy P, et al.	2019	Factors impinging on the education of Roma children in Ireland	Y	Ethnicity (Roma)	Ireland	Mixed methods	Social determinants of health	1, 3
29	Laird E, et al.	2020	To assess the vitamin D status from a selection of the Dublin population of South East Asian descent	Y	Nationality	Ireland	Quantitative	Non-communicable disease	4, 7
30	Ledoux, C., et al.	2018	Policy framework for migrants’ access to healthcare in Spain, Portugal and Ireland	Y	Country of residence	Ireland, Spain, Portugal	Qualitative	Healthcare policy	4
31	MacFarlane A, et al. (a)	2021	Adaptation of primary care services to improve cross-cultural communication by implementing the use of trained interpreters in general practice consultations.	Y	Habitual place of residence	Ireland	Qualitative	Communication barriers	5
32	MacFarlane A, et al. (b)	2020	To analyse how interpretation is used, funded and governed in primary care practices serving refugees in Australia, Canada, Ireland and the US.	Y	Refugees	Australia, Canada, Ireland and the US	Qualitative	Communication barriers	5
33	Mahon D, et al.	2023	Value of peer work in mental health, substance use, migrant health and homeless services	Y	Marginalised migrants	Ireland	Qualitative	Healthcare utilisation	2
34	Markey K, et al. (a)	2022	Migrant women at risk of or are experiencing perinatal mental illness	Y	Habitual place of residence	Ireland	Qualitative	Mental health	1
35	Markey K, et al. (b)	2018	Concerns and challenges experienced by undergraduate students and qualified nurses for caring for patients from diverse cultural, ethnic and linguistic backgrounds	Y	Nationality	Ireland	Qualitative	Healthcare workers	2
36	Mohan, G.	2021	To examine the health-care contact of children for whom their primary caregiver is foreign-born	Y	Nationality	Ireland	Quantitative	Healthcare utilisation	4,5
37	Murphy R, et al.	2021	Mental health experiences of African asylum seekers in Ireland.	Y	Asylum seeker	Ireland	Qualitative	Mental health	4
38	Nolan E	2023	International trained doctors with foreign nationality adjustment to working and living in Ireland	N	Nationality	Ireland	Quantitative	Healthcare workers	3
39	Noonan M, et al.	2018	Perinatal mental health problems in primary care.	N	Ethnicity	Ireland	Qualitative	Mental health	5
40	O’Brien KK, et al.	2023	Experiences of disability living with Long COVID	N	Race/ ethnicity	Canada, Ireland, UK and USA	Qualitative	Communicable disease	6
41	O’Donnell CA, et al.	2017	Training programme supporting the use of theory, in this case Normalisation Process Theory (NPT), in a multisite cross-country health services research study	Y	Asylum seeker & refugee	Austria, England, Greece, Ireland, The Netherlands and Scotland	Qualitative	Communication barriers	1, 5
42	O’Donoghue B, et al.	2021	Demographic characteristics of migrants presenting with a first episode of psychosis	Y	Nationality	Ireland	Quantitative	Mental health	5, 7
43	O’Reilly-de Brun M, et al.	2018	PLA techniques for data generation and co-analysis with multi stakeholder group	Y	Nationality	Austria, England, Greece, Ireland and The Netherlands, Scotland	Qualitative	Communication barriers	1, 5
44	O’Sullivan EJ, et al.	2021	Breastfeeding experiences and attitudes among Polish mothers living in Ireland	Y	Nationality	Ireland	Qualitative	Sexual and reproductive health	3
45	Palmer R, et al.	2019	Maternal health behaviours of non-Irish nationals during pregnancy and the influence of time living in Ireland	Y	Nationality	Ireland	Quantitative	Sexual and reproductive health	4, 5
46	Puthoopparambil SJ, et al.	2021	Levers and barriers to the provision of trained interpreters in healthcare settings in Ireland.	Y	Migrants not fluent in the language of their host country	Ireland	Qualitative	Communication barriers	5
47	P. Ryan et al.	2022	Women’s motivations in whether to disclose their sex work	N	Nationality	Ireland	Qualitative	Sexual and reproductive health	3
48	Schneider SM, et al.	2018	Ratings of Irish health services of the foreign-born in Ireland compared to those of Irish natives	Y	Nationality	Ireland	Quantitative	Health satisfaction	4
49	Scully H, et al.	2023	Determinants of vitamin D status in adults	N	Race, ethnicity	Ireland	Quantitative	Social determinants of health	4, 7
50	Smith K, et al.	2021	Issues around relationships with peers from the receiving community in the context of perceived difference and inequality.	Y	Nationality	Ireland	Qualitative	Mental health	3
51	Suko, P. I., et al.	2022	Coherence, health behaviour, acculturation, adaptation, perceived health, and quality of life of Croatian migrants living in Austria and Ireland.	Y	Nationality	Austria and Ireland	Quantitative	Health satisfaction	4
52	Swift, A., et al.	2021	Socio-emotional outcomes in children with disabilities and of migrant background using data from the Growing Up in Ireland study	Y	Nationality	Ireland	Quantitative	Social determinants of health	3, 4, 5
53	Thompson R, et al.	2022	Mental health crisis of GRT people	Y	Ethnicity	Ireland and UK	Qualitative	Mental health	7
54	Treston B, et al.	2022	Multisystem inflammatory syndrome in children (MIS-C) in the Republic of Ireland, in the context of all cases of COVID-19 in children	N	Race, ethnicity	Ireland	Quantitative	Communicable disease	4, 6
55	van den Muijsenbergh METC, et al.	2020	Impact of the NPT and PLA-guided implementation of guidelines and training initiatives to improve cross-cultural communication in primary care settings after a period of time.	Y	Nationality / ethnicity	Ireland, England, Greece, Netherlands	Qualitative	Communication barriers	5
56	van Gemert CE, et al.	2018	Chronic HBV in Ireland between 2004 and 2014 using routine surveillance data	N	Nationality	Ireland	Quantitative	Communicable disease	4, 6
57	Villani J, et al.	2021	Strategies adopted by community-health partnerships and NGOs to minimise the potential widening of Traveller and Roma health inequities during the COVID-19 pandemic	Y	Ethnicity	Ireland	Qualitative	Social determinants of health	6
58	Watters C, et al.	2022	Programme Refugees resettling in Ireland	Y	Refugees	Ireland	Qualitative	Mental health	3
59	White A, et al.	2019	Health status, satisfaction with life and the emotional well-being of Nigerian migrant parents living in Ireland and the Netherlands	Y	Nationality	Ireland and the Netherlands	Quantitative	Health satisfaction	4, 5
60	Zhou Q, et al. (a)	2020	Successful experiences of Chinese mothers living in Ireland who exclusively breastfeed	Y	Nationality	Ireland	Qualitative	Sexual and reproductive health	3
61	Zhou Q, et al. (b)	2020	Breastfeeding practices of the Chinese immigrants in Ireland	Y	Nationality	Ireland	Mixed methods	Sexual and reproductive health	3
62	Zhu, L.	2022	Religion as a variables between acculturation and mental health	Y	Nationality	Ireland	Quantitative	Social determinants of health	3

**Fig. 3:**
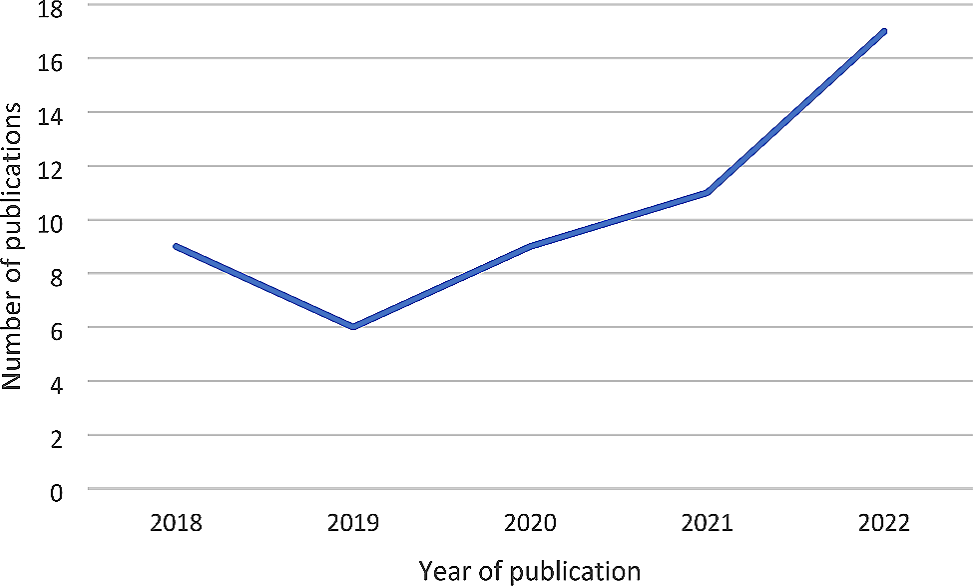
Number of migrant health studies included in this review by year of publication years (2017 and 2023 incomplete years and not included)

There has been an increase in studies over time from an average of five published, peer-reviewed studies per year in the previous review to an average of 10 per year in this review. Specifically, the volume of publications has increased over the last three years (see Fig. 3). 85% of included studies (*n* = 53) focused on migrant health research conducted exclusively in Ireland and 15% of studies (*n* = 9) were conducted elsewhere (other EU countries, the UK, Canada or the US) and included Ireland.

61% of studies (*n* = 38) declared their funding source and 39% of studies (*n* = 24) reported they did not receive funding or did not mention a funding source. Of those that declared funding; 76% (*n* = 29) received national funding and 24% (*n* = 9) received international funding.

### Characteristics of included articles

We found more quantitative (*n* = 34, 55%) than qualitative studies (*n* = 23, 37%) and there were also five (8%) mixed-method studies. Quantitative studies were mostly cross-sectional (*n* = 21, 62%). Six studies were cohort studies, six used a case series methodology and one was a diagnostic study. Of all 62 studies, 13% used a participatory research approach (*n* = 8).

Forty-three studies (69%) had a primary focus on the health of migrants and 19 studies (31%) had a secondary focus where the authors collected demographic data on migrant status or ethnicity as part of a broader study that investigated a specific health issue. 63% of these studies (*n* = 12) aggregated this demographic data and reported it in their findings. A further 6 studies collected demographic data on migrant status but did not report on it separately.

In terms of how studies classified or defined what a migrant is for the purposes of their research, the 43 studies whose primary focus was migrant health used categories including country of birth, citizenship, ethnicity, international protection status. The remaining 19 studies were general population studies that collected information on ethnicity, e.g. Roma, or racial categories such as Black, White, etc.

**Fig. 4:**
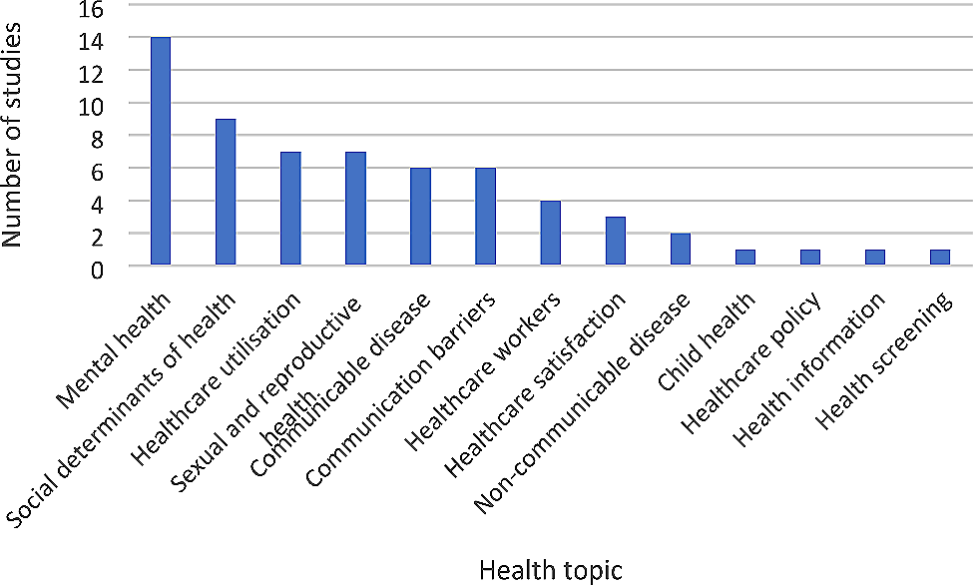
Health Topic of included studies (*n* = 62)

The primary health topic in each paper was identified by asking what health topic was the research designed to address? A summary of identified health topics is illustrated in Fig. 4.

The most commonly reported health topic was mental health (*n* = 14, 23%), including studies on migrant health workers that work in mental health services, participants experiences of depression, stress and perinatal mental health (#1, #11, #12, #13, #14, #19, #23, #34, #37, #39, #42, #50, #53, 58). The second most frequently reported health topic was the social determinants of health which reported on the wider socio-demographic factors that impact on health, e.g. migrants experiences of discrimination, education and housing factors (#17, #20, #21, #22, #28, #49, #52, #57, #62).

Other included studies focused on; sexual and reproductive health (#4, #5, #44, #45, #47, #60, #61); healthcare utilisation (#2, #10, #25, #26,#27, #33, #36); communicable disease (#3, #7, #16, 31, #40, #54, #56); communication barriers (#31, #32, #41, #43, #46, #55); healthcare workers (#8, #9, 35, #38); health satisfaction (#48, #51, #59); non-communicable disease (#15 and #29); child health (#24); healthcare policy (#30); health information (#18) and health screening (#6).

Studies were quality appraised using the tools and criteria already identified and categorised into low, medium or high quality; 65% of studies were assessed as high quality and 35% were considered moderate quality. No studies were assessed as low quality. The results of the quality appraisal are included separately as a supplementary file.

Collating / synthesising the data using WHO Strategy and Action Plan Strategic Areas.

**Fig. 5:**
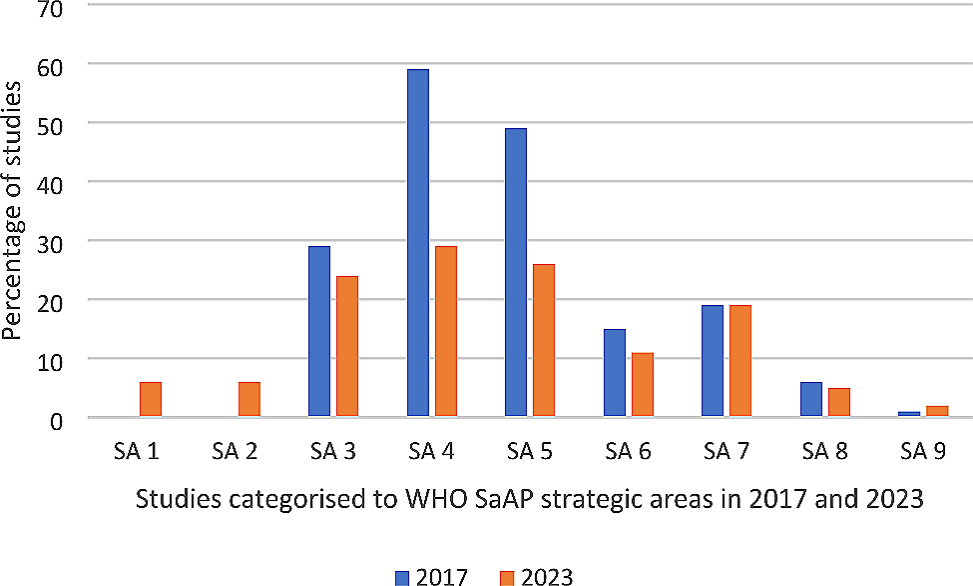
Categorisation of studies to WHO SaAP strategic area by year of review (2017, 2023)

Table 3 shows which study was categorised into the respective nine WHO SaAP strategic areas, and a comparison between the 2017 scoping review and our current review is shown in Fig. 5, with some studies coded to more than one strategic area.

**Table 3:** Categorisation of studies to Strategic Area and study ID.

Strategic Area	Number of studies coded to respective SA (%)	Article ID
SA1: Collaborative action	4 (6%)	#34, #28, #41, #34
SA2: Advocacy for the right to health	4 (6%)	#2, #17, #33, #35
SA3: Social determinants of health	15 (24%)	#1, #8, #9, #22, #38, #44, #47, #50, #58, #60, #61, #62, #28, #52, #16
SA4: Public health preparedness	18 (29%)	#10, #24, #25, #30, #37, #48, #51, #14, #52, #26, #27, #29, #36, #45, #49, #54, #56, #59
SA5: Strengthening health systems	16 (26%)	#7, #23, #26, #27, #31, #32, #36, #39 #41, #42, #43, #45, #46, #52, #55, #59
SA6: Communicable diseases	7 (11%)	#3, #40, #57, #6, #16, #54, #56
SA7: Non-communicable Diseases	12 (19%)	#4, #5, #11, #12, #13, #15, #19, #53, #14, #29, #42, #49
SA8: Health screening and assessment	3 (5%)	#20, #21,#6
SA9: Health information and communication	1 (2%)	#18

### Strategic Area 1: establishing a Framework for Collaborative Action

Four studies (6%) were classified under SA1 - #34, #28, #41, #34, three of which were qualitative and one was a mixed methods study, (compared to no studies in the previous review). Two studies (#41 and #43) were focused on collaborative action to improve the implementation of trained interpreters using Participatory Learning in Action combined with Normalisation Process Theory as the implementation framework. The third study (#34) also used participatory methods (online world café groups) to explore the collective roles of key stakeholders to support migrant women at risk of perinatal mental illness. The fourth study (#28) is a community-based participatory project with academic, NGO and government collaboration to improve Roma experiences in Ireland (this study was also coded to SA3).

This extensive network of collaborative partnerships encompassed a diverse range of stakeholders, involving senior primary care academics and researchers (#41), a coalition of migrants, general practice staff, and community interpreters (#43), as well as a collaboration with healthcare providers and community groups (#34), and engagement with both statutory and voluntary bodies (#28). It is noteworthy that all four studies classified under SA1 embraced participatory methodologies and in two studies facilitated the inclusion of migrants as peer researchers and stakeholders. This approach actively engaged migrants in dialogues pertaining to their healthcare experiences.

### Strategic Area 2: advocating for the Right to Health of Refugees, Asylum Seekers and migrants

Four very diverse studies - #2, #17, #33, #35 (6%), focusing on eliminating barriers to healthcare, were coded to SA 2, again compared to no studies in the previous review. Two of these studies had a qualitative design (#33, #35), one was quantitative (#2) and one was mixed method (#17). The importance of peer work with migrants (and other vulnerable cohorts) to improve healthcare utilisation by making care more culturally responsive was the focus of #33. This study found a disconnect between national policy and implementation on the ground. Concerns and challenges experienced by student nurses and qualified nurses caring for migrant patients and their feelings of uncertainty, lack of knowledge, their experiences of ethnocentric approaches, stereotyping and cultural factors within their own work places was the focus of #35. A third study (#2) looked at the barriers to healthcare experienced by migrants through their utilisation of healthcare compared to participants from Ireland and UK-born patients living in Ireland, with non-Irish and non-UK residents least expected to have attended a GP. The fourth study (#17) examined the relationship between stressful experiences such as exclusion from ordinary privileges and overt discrimination, with indicators of psychological well-being experienced by what the authors referred to as “visible” immigrant women of colour and “nonvisible” White immigrant women. This study found that “visible” immigrant women reported more experiences of discrimination than “nonvisible” immigrant women.

### Strategic Area 3: addressing the Social Determinants of Health

Similarly to the 2017 review where 29% of articles were categorised under SA3, fifteen studies (24%) reported on the social determinants of health; #1,#8, #9, #15, #22, #28, #38, #44, #47, #50, #52, #58, #60, #61 and #62. Six studies were qualitative in design, six were quantitative and three were mixed-methods studies.

Four studies related to occupation as a social determinant of health looking at **migrant healthcare workers** experiences of working across psychiatry (#1), midwifery (#9) and general practice (#8 and #38). All four studies examined satisfaction with working conditions and their plans to emigrate or return home as well as adjustment to working in Ireland (#38).

Two studies focused on the **mental health and well-being of migrants**; #50 explored the issues new Syrian migrants experience building relationships with peers in Ireland in the context of perceived difference and inequality and #58 examines the experiences of programme refugees in the West of Ireland. This study focused on the psychological well-being of refugees impacted by their experience living in a refugee camp and how this changed when they were resettled and acquired new social and cultural resources.

One participatory study (#28) explored the social determinants of health through the involvement of Roma peer researchers who identified that there were educational and legal implications for parents when they require their child to interpret their GP consultations with implications for school attendance.

### Strategic Area 4: Achieving Public Health Preparedness and ensuring an effective response

The largest number of studies (29%, *n* = 18) focused on achieving public health preparedness and ensuring an effective response - #10, #24, #25, #30, #37, #48, #51, #14, #52, #26, #27, #29, #36, #45, #49, #54, #56, #59, of which almost 90% were quantitative in design (*n* = 16). 78% of these studies had a primary focus on migrant health (*n* = 14) and 61% (*n* = 11) were coded to one other strategic area, most notably SA 5. SA4 was also the largest category of migrant health research in the previous scoping review at 59%.

Four of these studies reported on **healthcare utilisation and health preferences** of migrants and revealed the ways in which there is a lack of preparedness at present; three of which were by the same lead author. Study #26 examined Polish men’s lower utilisation of preventative healthcare and #25 and #27 looked at the health preferences and the negative utility value the men placed on their health. The fourth study (#36) found lower utilisation levels of general practitioner services for children of foreign-born residents in Ireland.

Two studies categorised under this strategic area focused on **communicable diseases** (#54 - Covid-19 and #56 - Hepatitis B virus) and were also categorised under SA 6 *preventing communicable diseases*.

Factors that drive **the health and emotional well-being** of migrant parents was the focus of #59, and findings reported in #10 found Syrian refugees experiencing high levels of anxiety due to unsatisfactory living conditions and unmet health needs.

**Satisfaction with the health service** was the main topic of the research conducted in #48 and #51; #48 looked at how foreign-born rate the Irish health system positively upon first arrival in the country and #51 examined adaptation and quality of life for Croatian migrants in Austria and Ireland. **Mental health** was the main focus of #14 and #37, specifically survivors of torture and asylum seekers awaiting a decision on their asylum application.

Three further studies focused on **maternal and child health**; #24, #45 and #52. Two studies addressed **vitamin D deficiency**; #29 and #49. An examination of **policy responses** to migrant health care was discussed in #30, which reported on the policy environment in Ireland, Spain and Portugal. Of the three countries involved in the policy review, Ireland is the only country which treats ethnic minorities and newly arrived migrants equally while Portugal and Spain only focus on the latter, which according to this review, makes Ireland’s migrant health policies highly inclusive.

### Strategic Area 5: strengthening Health systems and their resilience

Almost 26% of studies (*n* = 16) were categorised to SA5, in comparison to 49% in the previous scoping review. Fifteen studies had a primary focus on migrant health: #7, #23, #26, #27, #31, #32, #36, #41, #42, #43, #45, #46, #52, #55 and #59.

Nine of these studies are also coded to other strategic areas, namely SA4. Four studies (25%) were participatory and involved migrants as research stakeholders, representing service users of primary care services, to examine means of collaboration to strengthen the health system by improving communication and language barriers (#31, #43, 46 and #55). Study #39 focused on perinatal mental health and whilst it was a general population study, it also collected information on the health of migrants. Specifically, GPs perceived women from different ethnic and cultural backgrounds as reluctant to disclose their psychological distress and consequently GPs have concerns that they are not adequately understanding the concerns of women from ethnic and culturally diverse backgrounds.

Three studies were evaluations of participatory implementation programmes delivered through an EU research collaboration RESTORE 2011–2015 [39]; #41, #43 and #55. Three subsequent studies; #31, #32, #46 centred on the examination of **communication barriers** encountered by migrant populations in primary care and the use of interpreters.

### Strategic area 6: preventing Communicable diseases

Seven studies (11%) were categorised to strategic area 6; #3, #6, #16, #40, #54,# 56 and #57 (compared to 15% of studies in the previous scoping review). Two of the seven were qualitative in design (#40 and #57), four were quantitative (#6, #16, 54 and #56) and one applied a mixed-method design (#3). Three of the studies had a primary focus on refugee and migrant health (#3, #16 and #57) and four studies had a secondary focus (#6, 40, #57 and 56).

All seven studies focused primarily on specific communicable diseases, e.g. COVID-19 (*n* = 3), Tuberculosis (*n* = 1), Hepatitis B virus (*n* = 1), Measles (*n* = 1) and HIV (*n* = 1). Study #57 reported on a community health partnership to prevent the spread of COVID-19 within Roma communities and potential widening of health inequities during the initial response to the COVID-19 pandemic in Ireland. Mitigation interventions implemented include targeted public health measures, culturally sensitive communications, lobbying for policy change and social support.

Barrett (#3) also reported findings involving the Roma community, specifically a measles outbreak in 2016 and the factors which facilitated onward spread of disease. This report considered the strengths and weaknesses of ongoing measles control efforts in Ireland.

Of the two COVID-19 studies; #40 looked at the health-related challenges of adults living with long-COVID of which 25% were reported to be non-White. The second study (#54) reported on multisystem inflammatory syndrome in the context of paediatric COVID-19 infection and found that ethnicity appeared to have a major influence on incidence of MIS-C.

Van Gemert (#56) reported on the epidemiology of chronic Hepatitis B virus using routine data and found that amongst 2,696 chronic cases that the most commonly reported risk factor was being born in an endemic country (as either an asylum seeker or other immigrant). This study was also coded to SA4 *achieving public health preparedness and resilience*.

Finnegan (#16) reported on an outbreak of Tuberculosis in a Direct Provision centre in which 82 children (50% of whom were under 5 years of age), were considered close contacts of the index case and as a result at increased risk of developing disseminated TB and TB meningitis due to living in communal settings.

The final study that primarily focused on communicable disease was #6, which reported on a pilot project offering voluntary community-based HIV testing (VCBT) aimed at capturing data for at-risk populations not already attending clinical service in Ireland. This study was also coded to SA8 *ensuring ethical and effective health screening and assessment.*

### Strategic area 7: preventing and reducing the risks posed by Noncommunicable diseases

Twelve studies (19% - which is the same as the previous scoping review) were concerned with preventing and reducing the risks associated with noncommunicable diseases; #4, #5, #11, #12, #13, #14, #15, #19, #29, #42, #49 and #53. Over 90% were quantitative in design (*n* = 11). Six studies had a primary focus on migrant health and another six studies had a secondary focus. 25% of all studies were coded to other strategic areas, in particular SA 4. Half of all studies were concerned with **mental health**, psychological distress and trauma; #11, 13, #14, #19, 42 and #53. Of the six, two studies covered **child and adolescent mental health**. Cotter (#11) looked at a cohort of migrant youths 13 years old, as part of the Growing Up in Ireland study and their experience of stress and psychopathology, comparatively with their Irish counterparts. Haran (#19) reported on the practice of restrictive interventions (physical restraints and seclusion) in child and adolescent mental health facilities.

Four further studies reported on the mental health of adult migrants specifically; capacity and decision-making acuity of inpatients in a psychiatric facility (#13),;referrals to the national centre for victims of torture (#14); demographic characteristics of migrants presenting with first episode of psychosis in comparison to their Irish counterparts experiencing first episode of psychosis (#42); and the mental health needs of the Gypsy, Roma, Traveller population in Ireland and the UK (53).

A quarter of studies coded to SA7 covered **antenatal and sexual and reproductive health**; #4, #5 and #12. Bogdanet 2022a and 2022b (#4 and #5) looked at predicting gestational diabetes in pregnant women in their first and second trimester. Neither study collected primary data on migrants but did record ethnicity and tested ethnicity as a gestational diabetes risk factor. Both studies recommended future research with a larger, more diverse cohort to examine this association further. The third study (#12) examined a cohort of new mothers from the Growing Up in Ireland study and the prevalence of depression and depressive risk factors. The study found that depression symptoms were higher among ethnic minority mothers and being an ethnic minority mother was a primary determinant of not seeking treatment for depression.

Two further studies looked at the **Vitamin D deficiency**; #29 in a South East Asian population and #49 more broadly the determinants of Vitamin D deficiency of which non-White ethnicity was found to be significant. Both studies were also coded to SA4.

Finnegan (#15) was concerned with the risk factors for stroke and found that ‘originally not of Irish ethnicity’ constituted 9% of all stroke unit admissions to the acute stroke unit over a two-year period.

### Strategic Area 8: ensuring ethical and effective Health Screening and Assessment

Three studies were focused on health screening and assessment; #6, #20 and #21 in comparison to five studies (6%) in the previous scoping review. All three were quantitative in design and had a secondary focus on migrant health. One (#6) was also coded to SA 6. Two of the three studies reported on colour vision deficiency in 6- to 7-year-old school children in Ireland (#20, #21) and the third study reported on a voluntary community-based HIV testing pilot project aimed at collecting data from at-risk populations not already attending clinical service in Ireland (#6).

### Strategic Area 9: improving Health Information and Communication

One study; #18 reported on improving health information systems; a quantitative descriptive study which mapped the reporting of ethnicity and migration-related variables in national health and social care data collections in Ireland. Similarly one study was also categorised to SA 9 in the previous scoping review. The authors identified fourteen of 97 data collections with information on ethnicity. Country of birth was also collected in 10 of these 14 data collections. The authors found no routine recording of ethnicity in primary care, where the majority of healthcare is delivered, or for hospital inpatients, other than psychiatric inpatients.

## Discussion

### Summary of results

This review was designed to update an earlier scoping review of evidence about the health of migrants residing in Ireland conducted in 2017 [26]. The increase in the volume of research on migrant health in Ireland is notable, rising from an average of five published, peer-reviewed studies on migrant health research per year in the period 2000–2017 to an average of 10 studies per year in this review.

The percentage of studies on general population health with a secondary focus on migrant health has increased from 20% to 30% between the 2017 review and our current review. This indicates that more general population studies are beginning to include migrant status or ethnicity when collecting demographic data.

The previous scoping review found that the majority of studies (89%) were coded to either SA3, SA4 or SA5; the social determinants of health, public health preparedness and strengthening health systems. Although the number has reduced, our findings are consistent with the previous scoping review in that 70% of current studies (*n* = 49) were coded to either SA3 (*n* = 15), SA4 (*n* = 18) or SA5 (*n* = 16).

Ensuring public health preparedness and an effective response (SA4) remains a global priority for the WHO to be addressed over the next five years [5]. Therefore, it is encouraging to see a high volume of studies (29%) reporting on the health needs of refugees and migrants in the planning and development of public health services and policies. A wide variety of migrant health needs are being reported including mental health and well-being, sexual and reproductive health, communicable disease but also health preferences, satisfaction with health services and an examination of policy responses.

Over 26% of studies were concerned with strengthening health systems and their resilience (SA5) and specifically at optimising the delivery of healthcare to migrants experiencing language and cultural barriers in primary care. The levers and barriers to the implementation of trained interpreter services in Irish healthcare systems and different methodologies and theories to support the implementation process were reported on extensively. The need for such evidence is strongly emphasised in the first and second national intercultural health strategies (NIHS) in Ireland [14, 15].

Studies categorised to SA3 focused on the experiences of migrant healthcare workers working in psychiatry, midwifery and general practice and the environmental factors that contribute to their positive or negative associations with this work. Other studies included the experiences of Syrians building relationships with peers in Ireland and a programme for refugees resettled in the West of Ireland. Addressing health disparities and improving access to quality care for refugees and migrants extends beyond the capacity of health systems alone. Therefore, research on the influential nature of education, employment, social security, and housing to equitable access to healthcare is crucial. Study findings are compatible with evidence on the ground in the NGO sector, as described by TM, our community partner, who reports these factors have a profound impact on the health outcomes of migrants. Of particular importance to the NGO sector is that discernible attention is directed towards scrutinizing morbidity and mortality rates arising from social determinants of health within these migrant communities. This would reflect a commitment to comprehensively understanding and addressing the healthcare inequities that migrants encounter, which is aligned with the priorities set out in the first and second NIHS [15] and Global Action Plan 2019–2023 [40].

Strategic Areas 6 (preventing communicable diseases) and 7 (preventing and reducing the risks posed by noncommunicable diseases) consistently remain important topics for researchers, with a notable emphasis on noncommunicable diseases. Mental health and sexual and reproductive health emerge as the predominant and frequently studied domains within SA 7. This is notable as a recent scoping review of migrant health research in the UK found that mental health was the second highest most researched outcome for migrants in the UK [6]. The number of studies with a primary focus on the mental health or well-being of migrants or where the main health topic was mental health sits in contrast to a recent EU wide study that underscored mental health as a research priority across nine European countries [7].

The previous review found a scarcity of scientific research on collaborative action, advocating for the right to health for refugees and reported no studies relevant to either of those strategic areas. However, this review found an increase in studies focusing on SA1 and SA2 with eight (13%) studies focused on these strategic actions. In the second half of 2023, the WHO released two reports setting out the global research agenda on health, migration and displacement and promoting the health of refugees and migrants. Both reports reiterated that partnerships and interagency coordination and collaborative mechanisms remain a priority area of action [2] for WHO work around the globe and form an integral part of promoting equitable knowledge sharing and information transfer [5]. Our review reported on studies that developed innovative partnerships and collaborations between senior primary care academics and researchers; migrants, general practice staff, community interpreters, service providers, service planners from primary care; healthcare providers, community groups and NGOs, networks and associations who provide support to women and migrant communities. Interestingly two of the four studies coded to SA 1 used participatory methods to include migrants as peer researchers and stakeholders in dialogues about their healthcare.

Ethical and effective health screening, assessment and health information play a vital role in early detection and management of communicable diseases, public health threats and trust in the health system. However, this review found that there are few studies in this area. Studies coded to SA8 and SA9 were infrequently reported and there has been little change in the volume of research examining these strategic areas over the past six years. This finding points to a persistent gap in migrant health research in Ireland and a potential deficit in knowledge about prevention and early intervention through screening and assessment. It resonates strongly with the experiences of NGOs who report considerable resistance to breast cancer screening for example, due to possible stigma or fear. Conversely, NGOs see that some transient migrants without a GP remain excluded from screening programmes. IP and BoTP applicants are offered screening at accommodation centres, but those who have moved through the system, EU migrants and undocumented migrants can remain on the periphery of the health system and face challenges in connecting to screening programmes. It is also the experience of migrant NGOs that migrants without a Personal Public Service number (a unique identifier of individuals in Ireland) face exclusion from the health system, an issue that particularly affects the Roma community in Ireland and undocumented migrants. The situation is exacerbated by the limited attention paid to information systems on the health of refugees and migrants. Strengthening health information systems is a key priority within the global research agenda on health, migration and displacement and remains an issue for many countries across the globe [41].

The new Action Plan for Refugee and Migrant Health in the WHO European Region 2023–2030 [27] has 5 main action pillars, which could be used as a further benchmark when analysing the findings of this scoping review. Our findings suggest that Ireland must pay closer attention to Action Pillar 4: Strengthen Migration Health Governance and Evidence and Data-Driven Policy-Making in terms of future research in health screening, assessment, health information and communication.

### Meaningful participation of refugees and migrants

The attention given to participatory approaches and action research was noted in the first review of migrant health research in Ireland in 2001, specifically methodologies such as participatory action research and participatory learning and action that support migrants to engage in and contribute to the research with migrants rather than ‘on’ them [24]. This is in line with WHO guidance for robust evidence generation in the field [42].

Recommendations from the UK and Norwegian scoping reviews of migrant health research also claim that it is critical to meaningfully involve refugees and migrants from the research design to research dissemination in order to capture their healthcare priorities and meet their needs, and to ensure that the research process is not tokenistic or harmful [6]. This may require a ‘methodological shift’ but will lead to greater clarity on the health issues affecting migrants and further insights into the social determinants of health and health inequities [8]. This review identified eight studies (13%) that used participatory approaches; categorised to SA1 and SA 5. This is in contrast with the 2017 review, which identified only one study, categorised to SA 9. Further research to explore learnings from studies that used participatory approaches would be valuable to learn about, for example, what conceptual frameworks guided the research and how best to establish partnerships for meaningful involvement of refugees and migrants in research teams.

### Methodological critique

The strengths of our scoping review include a comprehensive overview of the published literature on migrant health in Ireland, rigorously following established scoping review guidelines with the addition of a quality appraisal for systematic and meticulous scoping of the literature. We replicated the use of the WHO SaAP and coding rules, which proved valuable for comparative and accumulative synthesis of evidence. The community consultation process provided an alternative voice from the perspective of migrant NGOs and is expected to strengthen the dissemination and veracity of the review’s findings [26].

There are some limitations to the scoping review including the exclusion of grey literature which would have provided additional evidence. Finally, it should be noted that even with careful development and refinement of coding rules, allocating a paper to one strategic area was not always straightforward because of cross-cutting aspects of research and/or migration health but consistency of coding was supported by regular team meetings.

## Conclusion

Migrant health research in Ireland is growing at a significant rate and generating an expanding evidence base for Irish policy makers. This is vital for fostering social inclusion, promoting health equity, and ensuring that healthcare services are responsive to the diverse needs of the population. However gaps persist particularly in research on health screening and assessment and health information and communication. This may lead to a potential deficit in knowledge about prevention and early intervention through screening and assessment as well as ongoing gaps in health information about refugees and migrants.

In line with Goal 4 of Ireland’s current National Intercultural Health Strategy [15] which calls for a robust evidence base to inform policy making, one planned action from this research is to set-up a national database cataloguing migrant health research in Ireland. This database will provide easy access to the growing evidence base and comprehensive analysis of health trends and demands within the migrant community, contributing to public health education as well as oversight of the state of the art. This will be a useful resource for refugees and migrants, researchers, policy makers, NGOs and community stakeholders when writing submissions to government about gaps in services for migrants, and for health service planners when developing evidence-based policy responses.

### Electronic supplementary material

Below is the link to the electronic supplementary material.

Supplementary Material 1

Supplementary Material 2

Supplementary Material 3

Supplementary Material 4

## Data Availability

The datasets generated and/or analysed during the current study are available from the corresponding author on reasonable request.

## Abbreviations

BoTP: Beneficiaries of Temporary Protection
CASP: Critical Appraisal Skills Programme
EU: European Union
GDP: Gross Domestic Product
GP: General Practitioner
GRIPP: Guidance for Reporting Involvement of Patients and the Public
ID: Identity
IP: International Protection
JBI: Joanna Briggs Institute
MMAT: Mixed Methods Appraisal Tool
NIHS: National Intercultural Health Strategy
NGO: Non-governmental Organisation
OSF: Open Science Framework
PRISMA ScR: Preferred Reporting Items for Systematic Reviews and Meta Analyses – extension for Scoping Reviews
SaAP: Strategy and Action Plan
SA: Strategic Area
WHO: World Health Organisation
