# Supplementary material for: An updated scoping review of migrant health research in Ireland
Source: BMC Public Health. 2024 May 28;24:1425. doi: 10.1186/s12889-024-18920-0 (PMC11134938; doi:10.1186/s12889-024-18920-0)
Supplement: Supplementary file 1 — Supplementary Material 1 [file 12889_2024_18920_MOESM1_ESM.docx]

**Updated Scoping Review of Migrant Health Research in Ireland**

**Additional file 1: Search Strategy – Embase Final Search**

| #12 | #11 AND 'Article'/it | SR search 10Apr | 127 | 2023-04-10 | 2023-04-10 |  |
| --- | --- | --- | --- | --- | --- | --- |
|  | #11 | #10 AND (2017:py OR 2018:py OR 2019:py OR 2020:py OR 2021:py OR 2022:py OR 2023:py) | SR search 10Apr | 223 | 2023-04-10 | 2023-04-10 |
|  | #10 | (asylum* OR refugee* OR migrant* OR migrat* OR emigrant* OR emigrat* OR immigrant* OR nomad* OR foreign* OR ethnic* OR displaced OR stateless OR 'state less' OR noncitizen* OR 'non citizen*' OR outsider* OR newcomer* OR 'newly arrived' OR 'new arrival*' OR 'recent entrant*' OR 'non national' OR 'non national':ab) AND 'health':ab AND 'ireland':ab  Collapse | SR search 10Apr | 1,087 | 2023-04-10 | 2023-04-10 |
|  | #9 | ('migrant':ta,ab OR 'refugee':ta,ab OR 'asylum seeker':ta,ab OR 'emigrants':ta,ab OR 'immigrants':ta,ab OR 'nomads':ta,ab OR 'foreigner':ta,ab OR 'ethnic group':ta,ab OR 'forced migrant':ta,ab OR 'statelessness':ta,ab OR noncitizen:ta,ab OR 'non citizen':ta,ab OR outsider:ta,ab OR newcomer:ta,ab OR 'newly arrived':ta,ab OR 'new arrival':ta,ab OR 'recent entrant':ta,ab OR 'non national':ta,ab OR 'non-national':ta,ab) AND 'health':ta,ab AND 'ireland':ta,ab AND [2017-2023]/py  Collapse | SR search 5Apr23 | 51 | 2023-04-05 | 2023-04-05 |
|  | #8 | ('migrant':ta,ab OR 'refugee':ta,ab OR 'asylum seeker':ta,ab OR 'emigrants':ta,ab OR 'immigrants':ta,ab OR 'nomads':ta,ab OR 'foreigner':ta,ab OR 'ethnic group':ta,ab OR 'forced migrant':ta,ab OR 'statelessness':ta,ab OR noncitizen:ta,ab OR 'non citizen':ta,ab OR outsider:ta,ab OR newcomer:ta,ab OR 'newly arrived':ta,ab OR 'new arrival':ta,ab OR 'recent entrant':ta,ab OR 'non national':ta,ab OR 'non-national':ta,ab) AND 'health':ta,ab AND 'ireland':ta,ab  Collapse | SR search 5Apr23 | 206 | 2023-04-05 | 2023-04-05 |
|  | #7 | #6 AND (2017:py OR 2018:py OR 2019:py OR 2020:py OR 2021:py OR 2022:py OR 2023:py) AND ('Article'/it OR 'Review'/it) | SR final search | 49 | 2023-04-03 | 2023-04-03 |
|  | #6 | (migrant:ab,ti OR refugee:ab,ti OR 'asylum seeker':ab,ti OR immigrant:ab,ti OR nomads:ab,ti OR foreigner:ab,ti OR 'ethnic group':ab,ti OR 'forced migrant':ab,ti OR statelessness:ab,ti OR migrat:ab,ti OR emigrant:ab,ti OR emigrat:ab,ti OR displaced:ab,ti OR 'non-citizen':ab,ti OR noncitizen:ab,ti OR outsider:ab,ti OR newcomer:ab,ti OR 'newly arrived':ab,ti OR 'new arrival':ab,ti OR 'recent entrant':ab,ti OR 'non national':ab,ti) AND health:ab,ti AND ireland:ab,ti OR (irish:ab,ti AND citizen:ab,ti)  Show full | SR final search | 221 | 2023-04-03 | 2023-04-03 |
|  | #5 | #4 AND (2017:py OR 2018:py OR 2019:py OR 2020:py OR 2021:py OR 2022:py OR 2023:py) | SR search | 37 | 2023-04-03 | 2023-04-03 |
|  | #4 | #3 AND ('Article'/it OR 'Review'/it) | SR search | 159 | 2023-04-03 | 2023-04-03 |
|  | #3 | (migrant:ab,ti OR refugee:ab,ti OR 'asylum seeker':ab,ti OR immigrant:ab,ti OR nomads:ab,ti OR foreigner:ab,ti OR 'ethnic group':ab,ti OR 'forced migrant':ab,ti OR statelessness:ab,ti OR migrat:ab,ti OR emigrat:ab,ti OR emigrant:ab,ti OR 'non-national':ab,ti OR 'recent entrant':ab,ti) AND health:ab,ti AND ireland:ab,ti  Collapse | SR search | 196 | 2023-04-03 | 2023-04-03 |
|  | #2 | #1 AND (2017:py OR 2018:py OR 2019:py OR 2020:py OR 2021:py OR 2022:py OR 2023:py) | SR search 3/4/23 | 62 | 2023-04-03 | 2023-04-03 |
|  | #1 | ('migrant'/exp OR migrant) AND health:ab,ti AND ireland:ab,ti | SR search 3/4/23 | 222 | 2023-04-03 | 2023-04-03 |
