# Supplementary material for: An updated scoping review of migrant health research in Ireland
Source: BMC Public Health. 2024 May 28;24:1425. doi: 10.1186/s12889-024-18920-0 (PMC11134938; doi:10.1186/s12889-024-18920-0)
Supplement: Supplementary file 2 — Supplementary Material 2 [file 12889_2024_18920_MOESM2_ESM.docx]

**Updated Scoping Review of Migrant Health Research in Ireland**

**Additional file 2: Quality Analysis**

| Study Design | Tool | No. of studies | Results (Low, moderate, high quality) |  |
| --- | --- | --- | --- | --- |
| Cohort | Newcastle Ottawa Scale | 6 | 6 High |  |
|  |  |  |  |  |
| Cross sectional | AXIS tool | 21 | 11 High 10 Moderate |  |
|  |  |  |  |  |
| Diagnostic | JBI checklist for diagnostic test accuracy | 1 | 1 High |  |
|  |  |  |  |  |
| Qualitative | Critical Appraisal Skills Programme (CASP) | 17 | 9 High 8 Moderate |  |
|  |  |  |  |  |
| Qualitative PHR | Guidance for Reporting Involvement of Patients and the Public (GRIPP 2) | 8 | 5 High 3 Moderate |  |
|  |  |  |  |  |
| Mixed-methods | Mixed Methods Appraisal Tool (MMAT) | 3 | 2 High 1 Moderate |  |
|  |  |  |  |  |
| Case series | JBI critical appraisal tool | 6 | 6 High |  |
|  |  |  |  |  |
|  | |  |  |  |
