# Supplementary material for: An updated scoping review of migrant health research in Ireland
Source: BMC Public Health. 2024 May 28;24:1425. doi: 10.1186/s12889-024-18920-0 (PMC11134938; doi:10.1186/s12889-024-18920-0)
Supplement: Supplementary file 3 — Supplementary Material 3 [file 12889_2024_18920_MOESM3_ESM.docx]

**Updated Scoping Review of Migrant Health Research in Ireland**

**Additional file 3: WHO Strategy and Action Plan (SaAP) Coding Rules**

**WHO Europe strategy and action plan for refugee and migrant health: coding rules for updated scoping review of refugee and migrant health research in Ireland, 2023.**

Developed by: Prof. Anne MacFarlane, Prof. Ailish Hannigan, School of Medicine, University of Limerick.

**_________________________________________________________________________________**

**The main objective and aims of a study are used to code it to one or more strategic areas. Examples of the types of studies coded to a strategic area are given. This is not an exhaustive list.**

**Strategic areas**

**1. Establishing a framework for collaborative action:** The aim is to promote and strengthen collaborative action on migrant health issues among international, national and local organisations and institutions. Studies that describe outcomes from collaborative action or studies that conduct methodological research on collaborative action.

**2. Advocating for the right to health of refugees:** The aim is to contribute factual and precise information to reduce discrimination and stigmatisation, and to eliminate barriers to health care for refugees and migrants. Studies that aim to describe interventions focused on legal and human rights to health, or provide evidence about discrimination and stigmatisation.

**3. Addressing the social determinants of health:** The aim is to build upon an adequate policy dialogue on the health of refugees, asylum seekers and migrants across all the involved government states and public. Actions include assessing how health opportunities and risks vary according to the social, economic and environmental determinants, including access to and security and density of housing, education, income and employment. Studies about employment conditions, the healthy migrant effect, legal status and health, the influence of country of birth, ethnicity and citizenship on breastfeeding practices. Includes studies about migrant workers (not working in the healthcare system).

**4. Achieving public health preparedness and ensuring an effective response:** The aim is to incorporate the health needs of refugees, asylum seekers and migrants in the planning and development of public health services and policies based on Health 2020. Studies that show evidence of migrants’ health needs (likely to have strong overlap with area 3).

**5. Strengthening health systems and their resilience:** The aim is to focus on the capacity to reach an agreement on the healthcare system competencies required to respond to the health needs of refugees and migrants, in a culturally sensitive way, with a particular emphasis on vulnerable groups. Studies about migrant health workers (working in the health system), language barriers, maternity care.

**6. Preventing communicable diseases:** The aim is to provide the necessary capability to focus on communicable diseases in transit and destination countries. Studies about preventing HIV, TB, malaria, infectious rashes.

**7. Preventing and reducing the risks posed by non-communicable diseases:** The aim is to establish that the needs of refugees and migrants form part of the national strategy for the prevention and control of non-communicable diseases. Studies about lifestyle factors (diet, smoking), health literacy, prevention of specific non-communicable diseases e.g. diabetes and health behaviours in pregnancy. Also includes articles focused on the mental health of migrants.

**8. Ensuring ethical and effective health screening and assessment:** The aim is to ensure that screening is risk-specific and evidence-based and serves the real interests of refugees, asylum seekers and migrants and the host population. Studies about screening of communicable and non-communicable diseases.

**9. Improving health information and communication:** The aim is to provide the adequacy, standardisation and comparability of records on the health of refugees, asylum seekers and migrants, to facilitate access to health information. Actions include promoting the inclusion of migrant variables in existing data collection systems and using innovative approaches, including surveys and qualitative methods, to collect data on refugees, asylum seekers and migrants. Studies about health information systems and records.

Source: *Modified from WHO, Strategy and Action Plan for refugee and migrant health in the WHO European Region. 2016 in Villaroel et al, 2017.*
